# Are Articulatory Settings Mechanically Advantageous for Speech Motor Control?

**DOI:** 10.1371/journal.pone.0104168

**Published:** 2014-08-18

**Authors:** Vikram Ramanarayanan, Adam Lammert, Louis Goldstein, Shrikanth Narayanan

**Affiliations:** 1 Signal Analysis and Interpretation Laboratory, Viterbi School of Engineering, University of Southern California, Los Angeles, California, United States of America; 2 Department of Linguistics, University of Southern California, Los Angeles, California, United States of America; The University of Chicago, United States of America

## Abstract

We address the hypothesis that postures adopted during grammatical pauses in speech production are more “mechanically advantageous” than absolute rest positions for facilitating efficient postural motor control of vocal tract articulators. We quantify vocal tract posture corresponding to inter-speech pauses, absolute rest intervals as well as vowel and consonant intervals using automated analysis of video captured with real-time magnetic resonance imaging during production of read and spontaneous speech by 5 healthy speakers of American English. We then use locally-weighted linear regression to estimate the articulatory forward map from low-level articulator variables to high-level task/goal variables for these postures. We quantify the overall magnitude of the first derivative of the forward map as a measure of mechanical advantage. We find that postures assumed during grammatical pauses in speech as well as speech-ready postures are significantly more mechanically advantageous than postures assumed during absolute rest. Further, these postures represent empirical extremes of mechanical advantage, between which lie the postures assumed during various vowels and consonants. Relative mechanical advantage of different postures might be an important physical constraint influencing planning and control of speech production.

## Introduction

Articulatory setting (AS) may be defined as the set of postural configurations (which can be language-specific and/or speaker-specific) that the vocal tract articulators tend to be *deployed from* and *return to* in the process of producing fluent and natural speech [1]–[4]. It is also variedly referred to as phonetic setting or organic basis of articulation or voice quality setting. A postural configuration might be, for example, a tendency to keep the lips in a rounded position throughout speech, or a tendency to keep the body of the tongue slightly retracted into the pharynx while speaking [5]. Historically AS has been the subject of linguists' intrigue, but due to the lack of reliable articulation measurement techniques, it has not been studied extensively until recently [6]–[9]. An important question in speech planning is the extent of control exerted by the cognitive speech planner as an utterance (read or spontaneous) progresses (by the term “speech planner,” we mean a cognitive control system that directs and regulates the behavior of the speech motor apparatus). In earlier work, we observed that articulatory settings assumed differ during rest positions, ready positions and read inter-speech pauses, and, in that order, exhibit a trend for *decreasing* variability and thus, a possible *increasing* degree of active control by the cognitive speech planning mechanism [9]. Further exploration of AS could have important implications for understanding the speech motor planning process, especially in models of motor planning following a ‘constraint hierarchy,’ i.e., a set of prioritized goals defining the task to be performed [10].

If speech motor control is optimized – in any sense of the term – it is reasonable to expect that key controlled postures have important mechanical advantages. Because AS represents a base posture for deploying speech articulators, it might ideally provide some mechanical optimality and/or advantage toward achieving speech motor tasks. Mechanical optimization can take many forms, depending on the situation and whether a dynamical (e.g., force/energy expenditure) or purely kinematical (e.g., duration of movements) perspective is being considered. Given the rapidity of motor actions associated with human speech, an important mechanical advantage might be the speed with which motor tasks can be achieved. Kinematic criteria of this kind have been quantitatively explored in a wide variety of mechanical systems – everything from simple levers to robot arms – as the *speed ratio*, which is the ratio of task space velocities (or the space of goal variables of motor control) to those in articulatory postural space (or the space of controll*able* variables) [11], [12]. Ratios with large numerical values are said to be mechanically advantageous because small changes in postures can result in relatively large changes toward tasks. Perhaps the simplest example of this situation is provided by a class two lever, which amplifies force and speed on different sides of the fulcrum according to the ratio of lengths of those sides. Indeed, amplification of force and speed are the same under the assumption of preservation of power from articulators to tasks: 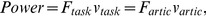
(1)where 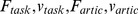 are the forces and speeds associated with the tasks and articulators, respectively. Following directly from this, it is possible to write more precisely that speed ratios are connected to mechanical advantage, as follows: 
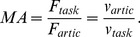
(2)


Equation 2 states the classical “Law of the Lever” discovered by Archimedes. More generally, mechanical advantage quantifies how the rate of change of input variables (in this case measured by the articulator speed, 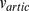) affects the rate of change of output/goal variables (as measured by task speed 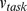). In other words, if a system has a large mechanical advantage, that implies that a small change in the space of articulators/controllable parameters results in a large change in the space of tasks/motor control goals.

**Figure 1:**
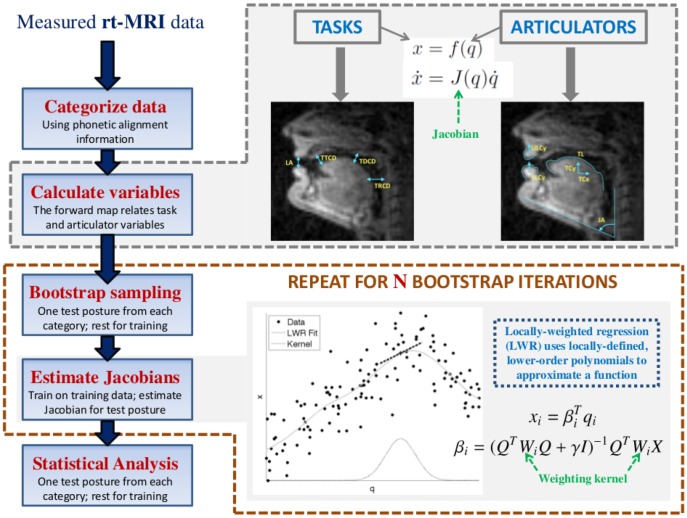
A schematic illustration of the analysis procedure.

With this in mind, the specific hypothesis of this study can be stated more precisely. The central hypothesis of this study is that postures assumed during pauses in speech, as well as speech-ready postures, have a much higher overall mechanical advantage or speed ratio when compared with postures at absolute rest. In other words, inter-speech postures allow for a larger change in the space of motor control tasks/goals for a minimal change in the articulatory posture space as compared to postures at absolute rest. This study is aimed at quantitatively testing this hypothesis using articulatory vocal tract data of real human speech data acquired with rtMRI [13]. Postures are described in terms of the spatial location of various speech articulators as well as the angle of the jaw, while task/goal variables are considered to be constriction degree at various points along the vocal tract. Please refer to the Methods section for further details. Figure 1 schematically depicts the experimental setup and flow of the paper.

In sum, we aim to answer the following question in this paper: are articulatory settings adopted during speech production more advantageous than rest positions in a kinematic sense, i.e., in facilitating efficient motor control of vocal tract articulators?

Recent advances in articulatory measurement techniques allow us to answer these questions more concretely. Some techniques that have been used to measure AS are X-ray microbeam or XRMB [6], electropalatography (EPG), electromagnetic articulography (EMA) [14] and ultrasound [8]. These techniques, although some are invasive, are able to capture articulatory information at high sampling rates. However, none of these modalities offer a complete a view of all vocal tract articulators, which is important for studying vocal tract posture. More recently, developments in real-time MRI have allowed for an examination of shaping along the entirety of the vocal tract during speech production and provide a means for quantifying the choreography of the articulators [13]. Although rt-MRI has an intrinsically lower frame rate than the other modalities, its superior spatial resolution as compared to other modalities makes it a better choice for an analysis of vocal tract posture.

## Materials and Methods

### Ethics Statement

This research was approved by the Institutional Review Board of the University of Southern California (UPIRB: https://oprs.usc.edu/upirb/). Participants provided written consent to participate in this study. Participant records/information were anonymized and de-identified prior to analysis.

### Direct and Differential Kinematics

Given a vector 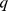, representing 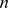 low-level articulator variables of the system, and a vector 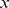, representing 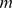 high-level task variables of the system, the relationship between them is commonly expressed by the direct kinematics equation, of the form: 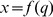
(3)where the function 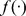 represents the forward map, a transformation from articulator to task space. In addition to the direct kinematics, it is also useful to consider the *differential kinematics* – which relate articulator space velocities to task space velocities. Of particular interested is modeling 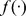 so as to facilitate derivation of the Jacobian matrix: 
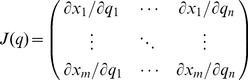
(4)


The Jacobian is a compact representation of the posture-specific 1*^st^*-order partial derivatives of the forward map. These can also be interpreted as speed ratios. The Jacobian allows us to conveniently write the differential kinematics equation in the following way: 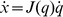
(5)


### Calculating Mechanical Advantage

**Figure 2:**
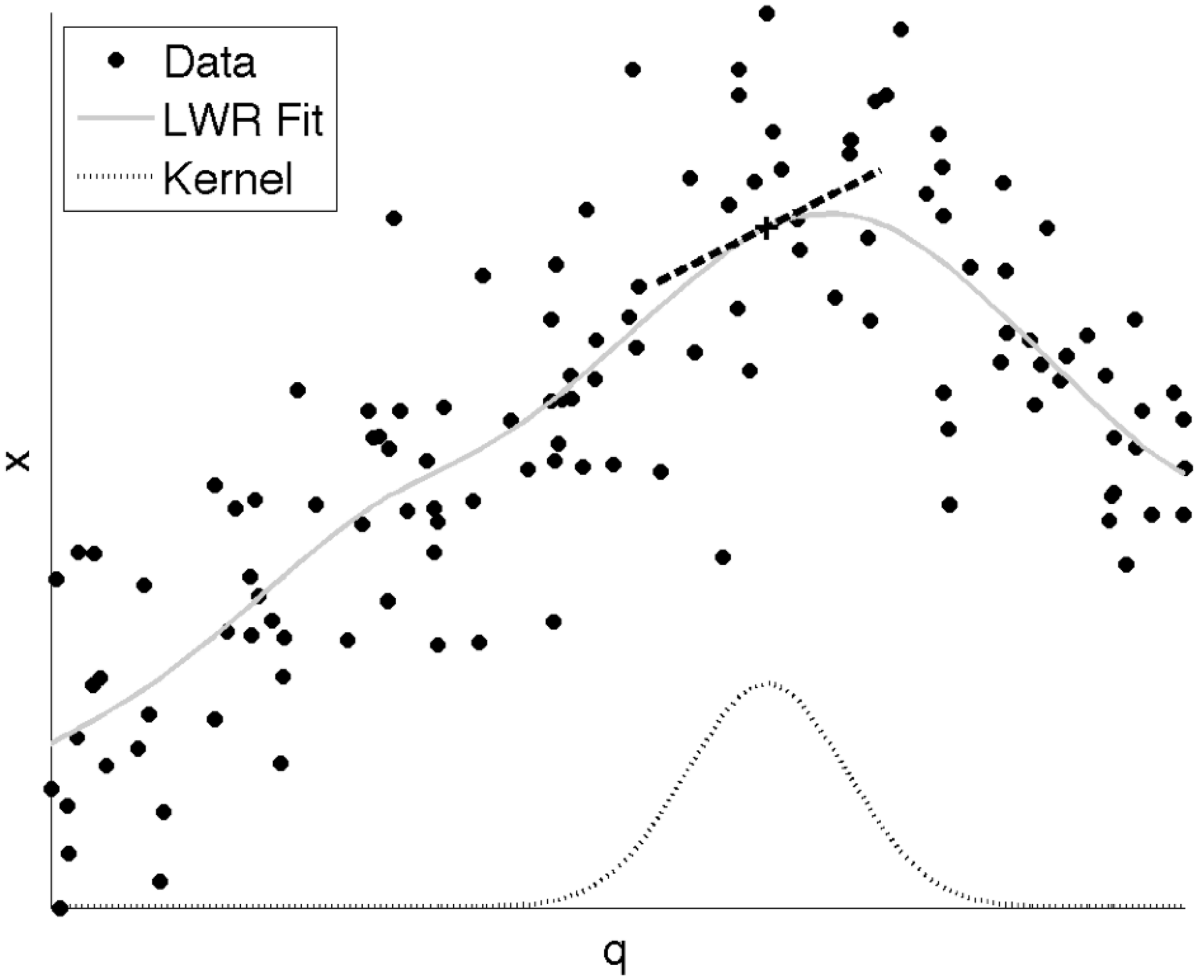
An illustration of modeling with Locally-Weighted Regression (LWR). For a particular point (black cross) a local region is defined in articulator space by a Gaussian-shaped kernel (gray dashed curve). A line is fit in the local region using a weighted least-squares solution, indicated by the black dashed line. The global fit is generated by repeating this procedure at a large number of local regions. The resulting fit can be quite complex (gray curve), and depends on the width of the kernel.

It is possible to accurately estimate kinematics of the vocal tract in a data-driven fashion. It was recently shown that Locally-Weighted Linear Regression (LWR) is useful for this purpose, producing accurate estimation and offering practical advantages [15]. LWR is a method that uses locally-defined, low-order polynomials to approximate globally nonlinear functional relationships. See Figure 2 for a graphical illustration of this function approximation technique. Training the model has a closed-form solution via the generalized least squares solution. The method also has few free parameters, making accurate training even more feasible. The result is a Jacobian matrix, relating the velocities of each task to each articulator. Each value in the Jacobian represents a speed ratio that could be used to characterize MA in the system. As an overall measure of MA, we computed the sum of squares of all Jacobian values.

### Example: Simulations on a Planar Robot Arm

In order to better understand and visualize how the notion of mechanical advantage might be useful in describing the posture of a motor system and understanding its control, we ran simulations on kinematic models of a multi-link planar robot arm with revolute joints. Recall that the mechanical advantage measure reflects how much change is observed in task/goal space for a unit change in articulatory space. Hence, we might expect that postures with a high value of mechanical advantage would be more “open”, since it might be easier to reach a given articulatory target therefrom with minimal change in the articulatory posture space, as compared to more constricted postures, which might possess a lower mechanical advantage value.

The simulated robot arm used in this application comprised three rigid links, three revolute joints and a single end-effector. The links were labeled from the base to the end-effector, and the lengths of these links were fixed to the values *l*_1_ = 1.0, *l*_2_ = 0.62 and *l*_3_ = 0.38. The corresponding joint angles were labeled in similar fashion as *q*_1_, *q*_2_ and *q*_3_. The Jacobian for this system can be written as: 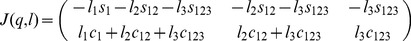
(6)where 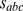 and 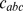 are shorthand for sine and cosine of angle summations. For example 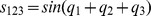 and 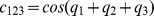.

Simulations involved manipulating the angles of the revolute joints to produce a wide variety of arm postures, and subsequently calculating mechanical advantage from the Jacobian at each of those postures. The joint angles that were considered for each joint spanned the range from 0 to 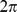 in increments of 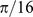. All possible combinations of angles for the three joints were considered, creating uniform coverage of joint-space. This large number of considered postures were sorted by their respective mechanical advantage values, and the highest- and lowest-valued postures were selected for visualization.

**Figure 3:**
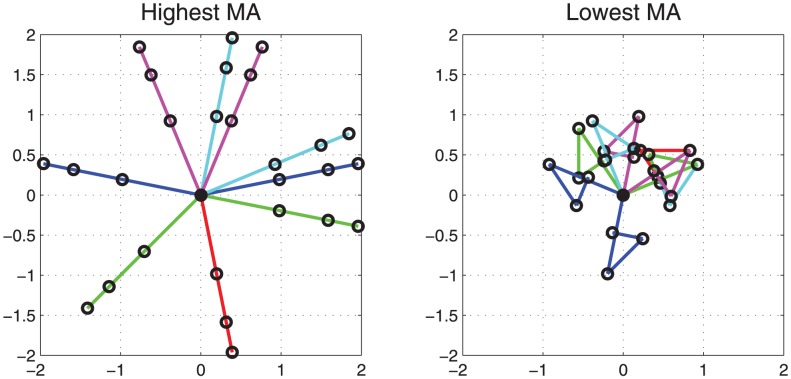
Planar robot arm configurations corresponding to the top eight (a) highest and (b) lowest average Jacobian values.

Figure 3 shows the model configurations corresponding to the top eight highest and lowest Jacobian values of the robot arm. We observe that postures with higher sum-squared Jacobian values are more open configurations, while more constricted, convoluted configurations have lower values of the same metric, in conformity with our expectations.

### Data

Five female native speakers of American English were engaged in a simple dialog with the experimenter on topics of a general nature (e.g., “what music do you listen to …”, “tell me more about your favorite cuisine …,” etc.) to elicit spontaneous spoken responses while inside the MR scanner. For each speech “turn,” audio responses and MRI videos of vocal tract articulation were recorded for 30 seconds and time-synchronized with the audio. The same speakers were also recorded/imaged while reading TIMIT shibboleth sentences and the rainbow passage during a separate scan. The spontaneous and read speech data represent the two speaking styles considered in this study. Details regarding the recording and imaging setup can be found in [13] and [16]. Midsagittal real-time MR images of the vocal tract were acquired with a repetition time of TR = 6.5 ms on a GE Signa 1.5 T scanner with a 13 interleaf spiral gradient echo pulse sequence. The slice thickness was approximately 3 mm. A sliding window reconstruction at a rate of 22.4 frames per second was employed. Field-of-view (FOV), which can be thought of as a zoom factor, was set depending on the subject's head size. Further details, and sample MRI movies can be found at http://sail.usc.edu/span.

### Extracting frames of interest from production data

Now that we have prepared and preprocesed our data – articulatory data with synchronized speech audio – our next step is to define pauses and phonetic categories of interest using the acoustic signal. In order to extract data frames corresponding to different categories of interest, a phonetic alignment of the data corpus was performed using the SONIC speech recognizer [17]. Based on this alignment, we first automatically extracted all frames of ISPs from the read and spontaneous speech samples [18]. Note that the SONIC speech recognizer uses a general heuristic of 170 ms between words before detecting and labeling a pause between those words. For the purposes of this study, we considered *only* grammatical ISPs, i.e., silent or filled pauses that occurred between overt syntactic constituents (including sentence end). In other words, we excluded pauses that were due to hesitation, word-search, etc., which do not appear to encode phonological information. Also note that phonetic context adjacent to these pause boundaries was not controlled. This was to allow for observation of articulatory setting characteristics during these pauses that were generic, i.e., not specific to any particular phonetic context. In addition, ‘speech-ready’ frames were extracted from each image sequence immediately before an utterance (a window of 100–200 ms before the start of the utterance as determined by phonetic alignment). Finally, the first and last frames of each utterance's MRI data acquisition interval were extracted as representatives of absolute rest position in the two speaking styles. Since subjects are cued to start speaking after they hear the MRI system “switch on,” it is assumed that the speaker's articulators will be in a “rest” position for the first frame of every acquisition. The phonetic alignment also allowed the extraction of frames corresponding to different phones categorized by manner and place of articulation.

Based on the phonetic alignments, the dataset was divided into 6 mutually exclusive, linguistically-meaningful categories: inter-speech pauses (ISP), absolute rest, speech-ready, vowels, obstruents (including stops, fricatives and nasals), and approximants (including liquids and glides).

### Computing task and articulatory posture variables

**Figure 4:**
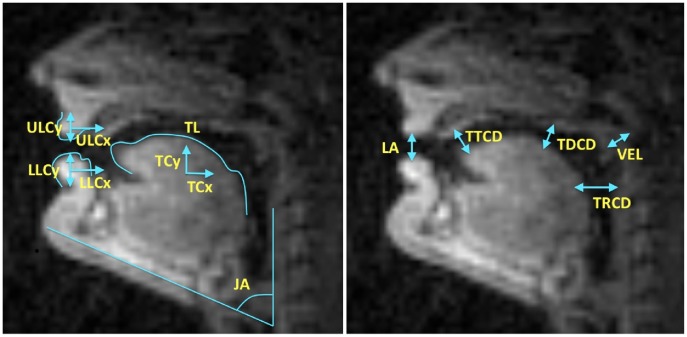
(a) Cross-distances in more detail (lip aperture (LA), velic aperture (VEL), and constrictions of the tongue tip (TTCD), tongue dorsum (TDCD) and tongue root (TRCD). (b) Articulatory posture variables – jaw angle (JA), tongue centroid (TC) and length (TL), and upper and lower lip centroids (ULC and LLC).

For all extracted frames for a given speaker, cross-distances were computed (namely, lip aperture, velic aperture, tongue tip constriction degree, tongue dorsum constriction degree and tongue root constriction degree) as representative *constriction task* variables, while geometric variables such as jaw angle, tongue length, and the centroids of the upper lip, lower lip and tongue were computed as representative *articulatory posture* variables. These variables were chosen for their compatibility with the frameworks of Task Dynamics [19] and Articulatory Phonology [20]. See Figure 4 for a visual schematic and [9] for more details on how these were extracted. Note that this is only *one* of many possible sets of variables we can choose as representative articulator and task variables. Other examples of possible task and articulator variables could be the set of formant frequencies and tongue shape parameters, respectively. The key point we want to make here is that the methodological framework is *generic* and *independent of the specific choice of varible set*. The *interpretation* of the results is, however, dependent on the specific choice of variable sets.

Each variable was then normalized by its range such that the transformed variable took values between 0 and 1. For example, if the tongue root constriction degree has a minimum value of 0.7 units and a maximum value of 2.5 units, then these values will correspond to 0 and 1 respectively after transformation. This allows us to compare variables across speakers while accounting for speaker-specific attributes, such as vocal tract geometry and gender. In addition, this type of transformation allows for more interpretable comparisons between different categories.

### Statistical Analyses

We now want to statistically quantify how the mechanical advantages of vocal tract postures assumed during different phonetic categories of interest differ from each other. For each category of interest (such as ISP, absolute rest, speech-ready, and so on) in a given speaker's data, Jacobian matrices were estimated using a bootstrapping procedure with 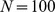 bootstrap samples. In each bootstrap iteration, a posture was randomly sampled from all the postures in that category to be used as a “test” posture. The LWR model was then fit to the rest of the data (training data), which was then used to estimate a Jacobian matrix for the test posture. The main free parameter that needs to be set in LWR is the standard deviation of the Gaussian kernel, which specifies the region of data space over which a linear regression will be performed. We set this parameter equal to 1.0 based on empirical experiments. Thus, at the end of the bootstrapping procedure we obtained 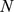 Jacobian estimates, and therefore 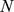 sum-squared-values of the Jacobian, for each category of interest (for a given speaker).

A non-parametric 2-way analysis of variance (Friedman's test) was performed to test the hypothesis that the medians of the different linguistic categories of interest were different. Note that in this case, the random factor is speaker (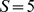 speakers) and there were 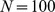 replicates in each block corresponding to the 100 bootstrap samples obtained earlier. Non-parametric Mann-Whitney U tests were also performed *post-hoc* for multicomparison tests (the data samples failed to pass Kolmogorov-Smirnov tests of normality. Hence, nonparametric tests were used here).

## Results

**Figure 5:**
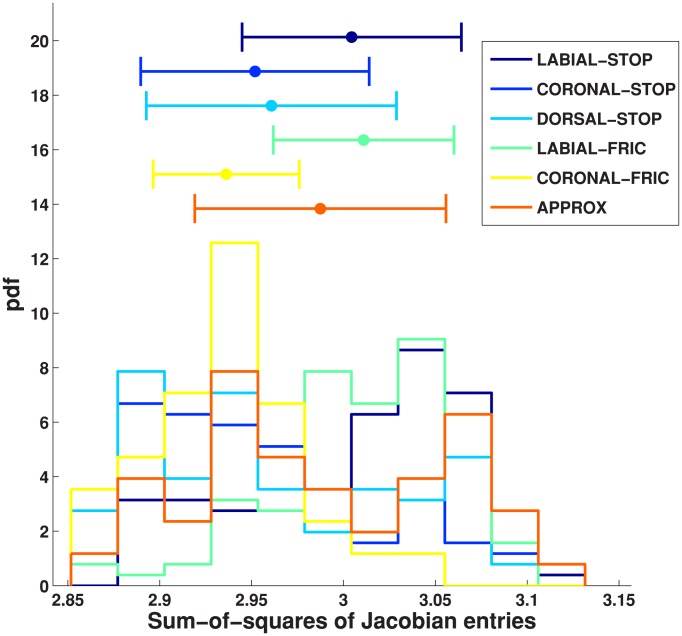
Histograms of the sum-squared values of Jacobians computed for different consonants on speaker Eng5's data.

The Friedman's test showed that the medians of the dependent variable (sum-squared values of Jacobian) were significantly different across the different categories of interest (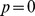). Table 1 shows the medians of the sum-squared values of all Jacobian entries, listed by linguistic category as well as speaker. We also tabulate the number of speakers for which we observed a pairwise difference in medians as determined by a post-hoc Mann-Whitney U test. Speech-ready postures are generally more mechanically advantageous than postures assumed during inter-speech pauses, which are in turn more mechanically advantageous as compared to postures assumed at absolute rest. Also postures during vowels are generally (4/5 speakers) more mechanically advantageous than obstruents. In addition, some interesting trends are exhibited among consonants. These trends are depicted in Figure 5 for speaker Eng5. Coronal fricatives were found to be less mechanically advantageous as compared to labial fricatives. This might be because coronal fricatives may require more task precision (or more stability) as compared to their labial counterparts, and the lower mechanical advantage value reflects *higher* stability. This, the reader will recall, is because the mechanical advantage measure reflects how much change is observed in task/goal space for a unit change in articulatory space.

**Table 1:** Medians of sum-squared values of the Jacobians tabulated by category and speaker (left).

Category	Medians of sum-squared Jacobian					No. of speakers with significant pairwise diffs.				
	Eng1	Eng2	Eng3	Eng4	Eng5	Rest	Ready	Vowel	Obs	App
ISP	4.19	5.63	7.41	6.95	5.17	3	5	4	5	3
Rest	4.14	5.55	7.35	6.96	5.14		5	4	4	3
Ready	4.23	5.55	7.44	6.99	5.18			5	4	4
Vowel	4.18	5.49	7.78	7.15	5.15				5	4
Obs	4.17	5.59	7.64	7.09	5.13					5
App.	4.25	5.47	7.38	6.95	5.17					

Also shown (right) for each pair of categories, are the number of speakers (out of 5) that returned a statistically significant difference on the Mann-Whitney U test for pairwise differences in medians at the 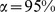 level. (Abbreviations: ISP  =  Inter-speech pause, Obs  =  Obstruent, App  =  Approximant).

## Discussion

This paper has motivated the importance of applying the notion of mechanical advantage to questions of interest regarding the speech production apparatus. MA is a basic mechanical concept with its origins in kinematic analysis but, to our knowledge, this concept has not been utilized for examinations in the domain of speech production. We presented a methodology for quantifying the mechanical advantage provided by different vocal tract postures by proposing methods to extract relevant task and articulator variables from rtMRI videos and for computing the Jacobian of the differential kinematic relationship between the two sets of variables.

We then explored a specific hypothesis of linguistic interest concerning articulatory settings which can be tested by quantifying and comparing the MA of different classes of vocal tract postures. We found support for the central hypothesis that postures assumed during inter-speech pauses (“articulatory settings”) are more mechanically advantageous than absolute rest postures with respect to speech articulation. In other words, articulatory setting postures afford large changes with respect to speech tasks for relatively small changes in low-level speech articulators. In the course of examining this hypothesis, we also find evidence that articulatory settings and speech-ready postures are more mechanically advantageous overall than other classes of vocal tract postures, including those assumed during different vowels, consonants and during absolute rest.

The observed differences between vowels and consonants is intriguing and suggests a way of grounding the traditional idea [21], [22] that consonant production is overlaid on a base formed by the production of vowels. In MA terms, the vowel could form an advantageous “launch-pad” for consonant constriction actions. Relatedly, MA could be one of the bases for the sonority hierarchy, which governs syllabification in languages. Differences among individual consonants and vowels may also provide some insight into their linguistic function, their acquisition, or their sensitivity to speech disorders. As an initial step in this direction, we uncovered some interesting patterns with respect to the mechanical advantage properties of certain fricatives and stops. For instance, we found that coronal fricatives were less mechanically advantageous (and thus, more stable) as compared to labial fricatives. Such observations have been attributed in the literature to non-linear quantal effects or saturation effects [23], [24] between motor control commands and articulatory movements, which might help detemine motor control goals. We postulate that the concept of mechanical advantage generalizes this notion of saturation effects, in that postures with low mechanical advantage are *stable*.

There are many other exciting avenues for future study. For instance, it is important to observe that the specific measures of mechanical advantage computed here (i.e., sum of squared of Jacobian values) are dependent on the choice of articulatory and task variables used for the differential kinematics estimation. This underscores the need for complementary ways of proceeding further: (i) finding an optimal set of task and articulatory variables with respect to MA and (ii) finding more expository measures of mechanical efficiency.
